# Role of Real-World Data in Assessing Cardiac Toxicity After Lung Cancer Radiotherapy

**DOI:** 10.3389/fonc.2022.934369

**Published:** 2022-07-19

**Authors:** Azadeh Abravan, Gareth Price, Kathryn Banfill, Tom Marchant, Matthew Craddock, Joe Wood, Marianne C. Aznar, Alan McWilliam, Marcel van Herk, Corinne Faivre-Finn

**Affiliations:** ^1^ Division of Cancer Sciences, School of Medical Sciences, Faculty of Biology, Medicine and Health, The University of Manchester, Manchester, United Kingdom; ^2^ Department of Radiotherapy Related Research, The Christie National Health Service (NHS) Foundation Trust, Manchester, United Kingdom; ^3^ Christie Medical Physics and Engineering, The Christie National Health Service (NHS) Foundation Trust, Manchester, United Kingdom

**Keywords:** real-world data, lung cancer, cardiac toxicity, radiation induced heart disease, heart dose constraints

## Abstract

Radiation-induced heart disease (RIHD) is a recent concern in patients with lung cancer after being treated with radiotherapy. Most of information we have in the field of cardiac toxicity comes from studies utilizing real-world data (RWD) as randomized controlled trials (RCTs) are generally not practical in this field. This article is a narrative review of the literature using RWD to study RIHD in patients with lung cancer following radiotherapy, summarizing heart dosimetric factors associated with outcome, strength, and limitations of the RWD studies, and how RWD can be used to assess a change to cardiac dose constraints.

## Introduction

Radiotherapy is the recommended treatment for approximately 50% of patients with cancer (1). Despite advances in radiotherapy techniques, some degree of radiation-induced toxicity remains inevitable. There is increasing evidence that cardiac toxicity is a concern in patients with lung cancer receiving radiotherapy and can occur earlier than previously thought. The impact of radiotherapy dose to the heart or sub-regions of the heart in patients with lung cancer receiving radiotherapy on overall survival (2–12), non-cancer deaths (13, 14), and incidence of cardiac events/deaths was recently demonstrated (2, 7, 8, 10, 15–20). However few studies have also incorporated the effect of baseline cardiac comorbidities or polypharmacy on radiation-induced heart disease (RIHD) and treatment outcome (8, 10, 15–20).

Randomized controlled trials (RCTs) are the gold standard method of providing evidence relating to efficacy and tolerability of treatment in the modern healthcare system (21). There are however many clinical scenarios, particularly in the radiotherapy setting, where there is no data available from RCTs and/or conducting RCTs is challenging, and therefore there is no clinical consensus on standard of care treatment. For example, older, frailer patients and those who present with higher level of comorbidities at diagnosis are well-known to be under-represented in RCTs (22), and as such, evidence to support decision making in these patient populations is often limited (23–25). Moreover, as radiotherapy advances rapidly, with much of its modification occurring through successive incremental technical developments rather than transformative step-changes, the impacts of such changes are challenging to test using classical RCTs. Furthermore, there is often a learning effect associated with new technologies, and hence a risk that results can quickly become outdated. Finally, there are common situations where trials would be difficult to design due to lack of clinical equipoise. For example when introducing image-guided radiotherapy (IGRT), many believed that imaging-based treatment would likely be superior to non-imaging-based treatment with regard to outcomes such as local control (26).

An alternative to RCTs is to provide evidence from the real-world setting that has the advantage of being more inclusive. Food and Drug Administration (FDA) defined Real-World Data (RWD) as “the data relating to patient health status and/or the delivery of health care routinely collected from a variety of sources” (27). Both the FDA and National Institute for Health and Care Excellence (NICE) are recognizing the importance of using routine data to evaluate how interventions tested in highly selected cohorts translate to the general population (28–30). In the field of cardiac toxicity, much of the information we have available comes from studies that used RWD. *Post-hoc* studies of cardiac toxicity from clinical trials of thoracic radiotherapy and retrospective studies of heart dosimetry and outcome can indeed be hypothesis generating e.g., on the interplay between baseline comorbidities and RIHD and the impact of cardiac dose on patient outcomes (2, 4–6, 16, 17, 31–33).

The aim of this article is to provide a narrative review of RWD studies in the field of RIHD in patients with lung cancer. By RWD studies, we mean studies that include data from patients that are not recruited to interventional experimental studies with specific inclusion/exclusion criteria. We summarize the existing literature derived from RWD, including data on heart dosimetric factors linked to outcome. Finally, we recap the strength and limitations of the RWD studies in this setting and describe how RWD can be used to evaluate a change to cardiac dose constraints.

## Real-World Data in the Context of Radiation Induced Heart Disease

### Clinical Context

Current cardiac dose constraints are mainly based on the Quantitative Analysis of Normal Tissue Effects in the Clinic (QUANTEC) and are mainly derived from radiotherapy in patients with esophageal cancer and lymphoma studies (34). In contrast to QUANTEC recommendations that mean heart dose should be kept below 15 Gy, Darby et al. presented a linear risk, no threshold model for major adverse cardiac events post-RT in a retrospective RWD case-control study that included >2000 individuals with breast cancer (30). In patients with lung cancer, survival remains poor; compared to patients with breast cancer, patients with lung cancer are typically older and have multiple comorbidities (35). The poor survival of patients with lung cancer taken together with the belief that RIHD has a long latency period based on data from the field of breast cancer and lymphoma (36, 37), have led to the underestimation of the risk of cardiac toxicity related to thoracic radiotherapy in patients with lung cancer. Moreover, higher cardiac dose exposure in patients with lung cancer may result in earlier onset of RIHD.

RTOG 0617 was the landmark clinical trial that kick-started worldwide awareness and interest in the field of RIHD in this setting. This RCT comparted a standard dose of thoracic radiotherapy (60 Gy in 30 fractions) to a higher dose (74 Gy in 37 fractions) delivered concurrently with chemotherapy +/- cetuximab (38). High-dose radiotherapy was associated with a higher risk of mortality, and multivariable models demonstrated that heart dose is an important prognostic factor for all-cause mortality (2). However, specific heart toxicity endpoints were not recorded in the trial, therefore the association of dose with RIHD or cardiac death could not be assessed. Wang et al. subsequently presented an analysis of pooled data from six lung cancer dose-escalation trials with endpoints for symptomatic cardiac death (15). In competing risk-adjusted cumulative incidence curves, for cardiac death, the impact of higher mean doses to the heart was shown. Patients receiving greater than 20 Gy mean heart dose were more than twice as likely to experience death due to a cardiac cause than patients with a mean heart dose of 10 Gy or less.

These *post-hoc* analyses of RCT data stimulated interest in the field of RIHD in lung cancer and researchers have since sought to supplement this evidence using RWD. Following the publication of RTOG 0617, it has been recognized that the latency time for RIHD in patients with lung cancer is much shorter than other thoracic cancers including patients with lymphomas and breast cancers who typically develop RIHD at least 5 years after radiotherapy (37, 39–42).

There is also an appreciation that the physiology of RIHD is complex. The heart consists of several connected anatomical sub-structures, each of which could have an associated radiotherapy dose response to radiation. Identifying the structures with the strongest association with RIHD is challenging due to the proximity of these sub-structures, meaning that the radiation dose between neighboring regions will be highly correlated. Despite these challenges, studies based on RWD have identified dose to sub-regions of the heart more strongly associated with patient outcomes. McWilliam et al., using RWD and a voxel-based data mining approach, reported radiation dose to the base of the heart had the greatest impact on survival in patients with lung cancer treated with radical radiotherapy (3). This region was further validated in patients with stage I non-small cell lung cancer (NSCLC) treated with stereotactic body radiation therapy (SBRT). Stam et al. analyzed the dose to cardiac sub-structures using a template anatomy, identifying the superior vena cava and left atrium as most strongly associated with non-cancer death in patients with early-stage NSCLC receiving SBRT (13). Similarly to Darby et al’s study in patients with breast cancer (36), in a RWD nested case-control study, Abravan et al. showed a linear relationship between mean dose to a region located at the base of the heart and cardiac-related deaths in patients with lung cancer treated with radical radiotherapy (43). No threshold has been identified, however, and whether such a relationship is linear or threshold based still remains to be understood. **Table 1** shows the results of studies utilizing RWD to investigate associations between heart dosimetric parameters and outcome in patients with lung cancer. Of note, most studies in lung cancer investigate the link between outcomes and planned dose (as opposed to delivered dose). However, the set-up uncertainties and anatomical motion impacts the dose received by the heart and heart sub-structures, and thereby the risk of RIHD (44).

**Table 1:** Summarizing RWD studies suggesting associations between heart dosimetric factors and outcome of patients with lung cancer in multivariable models.

Authors, year	Patient stage, population (n)	Study Institution	Correlation between heart dosimetric factors, other heart related factors and outcome in multivariable analysis
Speirs et al, 2016 (2)	Stage II-III NSCLC (416)	Single institution, Siteman Cancer Center/Barnes Jewish Hospital	Heart V_50_ associated with OS and cardiac toxicity
Mcwilliam et al, 2017 (3)	Stage I-IV Lung cancer (1101)	Single institution, The Christie	Mean dose to the identified region located in the base of the heart associated with OS
Stam et al, 2017 (13)	Stage I-II NSCLC (803)	Multi-institutional	Maximum dose on the left atrium and dose to 90% of the superior vena cava associated with non-cancer death
Stam et al, 2017 (4)	Stage II-III NSCLC (469)	Single institution, NKI	Heart V_2_ associated with OS
Wang et al, 2017 (15)	Stage III NSCLC (112)	Multi-institutional	MHD, heart V_5_, heart V_30_, and left ventricle V_5_ associated with CE in patients with IHD or high WHO/ISH risk scores. MHD ≥ 20 Gy higher rate of CE No association between OS and heart dose
Dess et al, 2017 (16)	Stage II-III NSCLC (125)	Multi-institutional	MHD and PCD associated with higher CE
Vivekanandan et al, 2017 (5)	Stage IIB-III NSCLC (78)	University of Oxford	ECG changes at 6 month and left atrium dose > 64 Gy associated with OS
Ning et al, 2017 (17)	Stage I-IV NSCLC (201)	Single institution, MD Anderson Cancer Center	Heart V35 >10% and PCD associated with PCE
Chun et al, 2017 (6)	Stage III NSCLC (482)	Multi-institutional	Heart V40 associated with OS
Yegya-Raman et al, 2018 (18)	Stage II-IV NSCLC (140)	Single institution, Rutgers Cancer Institute	MHD and baseline coronary artery disease associated with symptomatic CE
Wong et al, 2018 (14)	Stage I-II NSCLC (189)	Single institution, Princess Margaret Cancer Centre	Max dose (per 100 Gy) to left and right ventricle associated with non-cancer deaths
Xue et al, 2019 (7)	Stage I-III NSCLC (94)	Multi-institutional	MHD, hart V5, V55, pericardial mean dose, V5, V30, and V55 associated with PCE Pericardial V30 and V55 associated with OS
Atkins et al, 2019 (8)	Stage II-IIIB NSCLC (748)	Single institution, Dana-Farber Cancer Institute/Brigham and Women’s Hospital	MHD (≥10 Gy) associated with MACE and OS
Mcwilliam et al, 2020 (9)	Stage I-IV Lung cancer (978)	Single institution, The Christie	Max dose to the combined cardiac region including right atrium, right coronary artery, and ascending aorta associated with OS
Abravan et al, 2020 (19)	Stage II-III Lung cancer (1243)	Single institution, The Christie	Mean dose to the identified region overlapping with right atrium (≥10 Gy) associates with cardiac related death in patients without PCD
Atkins et al, 2021 (10)	Stage II-III NSCLC (701)	Single institution, Dana-Farber Cancer Institute/Brigham and Women’s Hospital	LAD coronary artery V15≥ 10% associated with MACE and OS, particularly in patients without CHD. Left ventricle V15 ≥ 1% associated with MACE in patients with CHD.
Shepherd et al, 2021 (11)	Stage I-III NSCLC (285)	Single institution, MSKCC	Heart V8 associated with OS
Abravan et al, 2021 (20)	Stage II-III Lung cancer (1218)	Single institution, The Christie	Mean dose to LAD associated with cardiac hospital admission and cardiac related death in patients without diagnosed PCD
Abravan et al, 2022 (43)	Stage II-III Lung cancer (2488)	Single institution, The Christie	Mean dose to cardiac avoidance region (superior vena cava, right atrium, aortic root, and proximal segments of the coronary arteries) linearly associated with the increase in the risk of cardiac related death

NSCLC, non-small cell lung cancer; OS, overall survival; MHD, mean heart dose; IHD, ischaemic heart disease; WHO/ISH, world health organization/international society of hypertension;CE, cardiac events; ECG, electrocardiogram; PCD, pre-existing cardiac disease; PCE, pericardial effusion; MACE, major adverse cardiac events; LAD, left anterior descending coronaryartery; CHD, chronic heart disease.

A complementary study from Manchester, again using RWD, investigated the impact of residual set-up errors on patient outcomes. For each fraction of radiotherapy, the patients’ positioning is checked with an on-board cone-beam CT scan. Any positional differences in the tumor can be corrected; however, these corrections can result in shifting the radiation dose towards or away from the heart. Over the full treatment course, this may result in an individual patient receiving a higher or lower dose to the heart than planned. In patients with stage III NSCLC, when the dose was moved in the direction of the heart (likely increasing the heart dose), patients had worse overall survival in multivariable analysis (45). The same effect was seen in patients with early-stage NSCLC treated with SBRT (46). Further analysis of the dataset of patients with stage III NSCLC investigated the dose differences due to these positional changes and identified a region in the base of the heart where the changed dose was most strongly associated with early mortality (47). Importantly, variation in residual set-up errors can be considered as random and compared to a natural experiment with no obvious correlation with other clinical or patient characteristics, allowing a causal relationship between the dose response to the base of the heart and risk of death to be inferred.

In addition to the studies in the field of lung cancer, a number of RWD studies have been reported on the impact of dose to heart or heart sub-structures on risk of cardiac events in patients with other thoracic tumors. For example, in patients with esophageal cancer, despite high level of competing risk, association between heart dose and cardiac events (48, 49), key coronary substructures (namely left anterior descending coronary artery [LAD]) dose and incidence of major coronary events (50) have been reported. In patients with breast cancer, association between mean heart dose and major coronary events (36), left ventricle dose and cardiac events (51), and LAD dose and increased requirement for coronary intervention in mid LAD (52) have been reported. However, it should be noted that heart exposure from tangential fields during breast radiotherapy only affects a small section of the heart compared to the exposure observed during thoracic radiotherapy for lung cancer (where one or more beams traverse the heart). Therefore, difference in dose distribution within the heart, baseline comorbidities, and age at diagnosis may partly explain why RIHD is an acute event in patients with lung cancer as opposed to a late event in patients with breast cancer.

### Baseline Cardiac Conditions

Identification of the burden and severity of cardiac comorbidities is important to personalize cardiac sparing in patients with lung cancer treated with thoracic radiotherapy. It has been established that comorbidities are an important predictor of early mortality (53). Indeed, about 75% of patients with lung cancer have known comorbidities at diagnosis with the most common being cardiovascular disease, chronic obstructive pulmonary disease, and diabetes (54–57). An area of interest in the field of cardiac toxicity in lung cancer is the impact of pre-existing cardiac condition on the risk of RIHD, given that a quarter of patients with lung cancer will present with a cardiovascular disease at diagnosis (55, 58, 59).

In a retrospective cohort of 1155 patients with lung cancer, Tammemagi et al. identified multiple comorbidities in two-thirds of patients, including 18 comorbidities that demonstrated stronger associations with early mortality than age, gender, or smoking (53). A United States National Cancer Institute Surveillance, Epidemiology and End Results (SEER) database RWD study of patients aged over 65 years with small cell lung cancer (SCLC) found that patients who had a cardiac event (acute myocardial infarction, cardiomyopathy, arrhythmia, heart failure or pericarditis) in the 12 months prior to treatment had an increased incidence of cardiac events following chemoradiotherapy (60). The rate of cardiac events was 55.4% in the year following radiotherapy in patients who had a previous cardiac event compared to 28% in those who had not had a previous cardiac event. A similar study in patients with NSCLC found an increased mortality in patients with known cardiac comorbidities following thoracic radiotherapy (59). Even when patients with cardiac comorbidities were excluded from the analysis, there was still an increase in cardiac events following radiotherapy in patients with multiple non-cardiovascular comorbidities (61).

Wang et al. paired World Health Organization/International Society of Hypertension (WHO/ISH) risk score with dosimetric parameters on multivariable analysis and found that patients with a high 10-year risk of a cardiovascular event had a significantly higher rate of cardiac events after radiotherapy (15). The WHO/ISH risk prediction charts not only indicate the risk of ischemic heart disease but also stroke and are only applicable to patients who have not yet had a cardiovascular event. Therefore their use is limited in a population of patients with lung cancer, over 25% of whom will have a history of a cardiovascular event (62). To overcome this issue, Dess et al. used the Framingham risk score, which predicted a patient’s risk of myocardial infarction or death from ischemic heart disease in a cohort of 71 patients with lung cancer and without pre-existing cardiac disease treated with dose-escalated radiotherapy (16, 63). This *post-hoc* analysis did not find any correlation between Framingham risk score and ≥ grade 3 cardiac event.

In a retrospective study of 748 patients who had radiotherapy for stage II-III NSCLC, Atkins et al. showed not only that patients with cardiac comorbidities had an increased rate of major adverse cardiac events following treatment compared with those without cardiac comorbidities, but also that mean heart dose ≥ 10Gy was associated with increased incidence of major adverse cardiac events (cardiac death, unstable angina, and myocardial infarction) in patients without a history of ischemic heart disease (8). They further reported that mean heart dose is not a suitable surrogate for LAD dose and the risk of major adverse cardiac events in the sub analysis of the same lung cancer cohort (10).

By having access to cause of death from Public Health England data, and hospital admissions from Hospital Episode Statistics, and utilizing voxel-based data mining, Abravan et al. investigated how radiotherapy dose in the thorax relates to cardiac-related death taking into account patient pre-existing cardiac conditions, using RWD from 1243 patients with lung cancer (19). Fine and Gray competing risk regression for cardiac-related death, with other causes of death as a competing risk, showed an increase in the risk of cardiac-related death in patients with pre-existing cardiac disease. Voxel-based data mining identified a region overlapping with the right atrium where dose was significantly higher in those patients who died due to a cardiac cause. Multivariable analysis suggested that radiotherapy dose to this region has the highest impact on cardiac-related death only in those patients without diagnosed cardiac conditions prior to treatment. In a further study, Abravan et al. studied the risk of cardiac hospital admission after radiotherapy and dose delivered to cardiac sub-structures in 1218 patients with lung cancer with no known pre-existing cardiac disease (20). Multivariable analyses showed that mean LAD dose correlates with both cardiac admission post-RT and cardiac-related death. Cardiac admission post-RT also correlates with cardiac-related death in the model including mean LAD dose. It is suggested that significance of LAD dose alongside cardiac admission in predicting cardiac-related death may point to undiagnosed cardiac disease in this population.

Calcifications are one established predictor for cardiovasular events (64–68) and are directly measurable from the CT scan acquired for planning a patient’s treatment. Utilizing RWD, Abravan et al. observed an association between the volume of calcifications found on the planning 4DCT scan and cardiac comorbidity scores obtained from Adult Co-Morbidity Evaluation (ACE-27) in 334 patients with lung cancer treated with SBRT (69). Multivariable models showed that the volume of calcification is an independent predictor of patient survival. Furthermore, for 428 patients, a deep-learning model was applied to identify calcifications from planning CT scans and stratify into low- and elevated-risk groups. Patients in the high-risk group were found to have an increased risk of all-cause mortality in the multivariable model (70).

### Other Toxicities Related to Heart Radiotherapy Dose: Lymphopenia

Other toxicities can also result from heart irradiation and further affect the outcome of patients with lung cancer. Incidence of lymphopenia (a drop in lymphocyte counts), has for example been reported following thorax irradiation. Severe lymphopenia has been shown to be associated with worse outcome in patients with lung cancer who received radiotherapy as part of their cancer treatment (71, 72).

Few RWD studies have addressed the effect of heart irradiation on lymphopenia and outcome. Ladbury et al. reported that higher radiotherapy dose to the “host immune system,” defined as a function of mean heart dose, mean lung dose, mean body dose, and number of fractions, was associated with overall survival in 117 patients with stage III NSCLC (72). Abravan et al. utilized a large cohort (>900) of patients with lung cancer receiving curative-intent radiotherapy and studied which organs are responsible for severe lymphopenia during radiotherapy when irradiated. Using voxel-based data mining, results showed an association between thoracic vertebrae V20, mean lung dose, mean heart dose and grade 3 or higher lymphopenia (71). Authors further showed that lymphopenia is an independent predictor for OS in both SCLC and NSCLC. Local irradiation to heart and lung affects circulating lymphocytes in the blood pool, which may explain one important mechanism of lymphopenia. Another study by Zhao et al. showed worse OS for 76 early-stage patients with lung cancer who developed grade 2 or higher lymphopenia after SBRT. A negative association between heart V5 and total lymphocyte count after SBRT was further indicated (73).

Evidence is emerging that both cardiac toxicity and lymphopenia are associated with cardiac irradiation, and further work is required to elucidate the relationship between toxic heart dose, lymphopenia, and patient outcome.

## Discussion

### What are the Strengths and Limitations of Real-World Data?

RCTs have the advantage of ensuring high internal validity in a way that observed effects are the result of the tested intervention. They provide high quality data but often they require additional patient procedures incurring greater expense or burden for patients. The downside of RCTs is that they lack generalizability. RCTs can be subjected to selection bias and hence may not accurately represent the patient population of interest (74, 75). Moreover, RCTs are often expensive and there are situations where they can be impractical, such as evaluation of technological advances in radiotherapy (26). In the field of radiotherapy-induced cardiac toxicity, given the accumulating evidence on the impact of dose to specific anatomical regions of the heart, it is becoming increasingly difficult to argue equipoise for the evaluation of new dose limits through classical RCTs. In such situations, the application of RWD can provide an alternative to conventional RCT evidence. Whereas much of the RWD evidence discussed above comes from retrospective observational studies, RWD is not only synonymous with this approach but can also be used prospectively to study the impact of new interventions in pragmatic trial designs (76).

It is however important to acknowledge that RWD studies have known limitations, primarily the risks of bias introduced by missing or incorrectly recorded data, and the inherent risk of unmeasured confounding in non-randomized datasets. In RIHD studies for example, target volume and location may influence not only the dose to the heart but also clinical outcome (survival) which can lead to false association in real-world research and may affect the validity of evaluations of interventions (77). In addition, dose exposure to one sub-structure in the heart is often co-linear with dose to another nearby sub-structure, such that the selection of which sub-structure or dose threshold is responsible for damage is usually done on statistical considerations, which is unlikely to reflect the underlying biology.

### How to Achieve High Quality Real-World Data Research?

There are concrete steps that can be taken to improve the quality of the RWD required to study RIHD. For example, heart or heart sub-structure segmentations are essential to successfully derive high-quality and meaningful evidence to inform decisions in the clinic. However, retrospective contouring of structures or sub-structures of interest is not realistic. The manual contouring of heart sub-structures on routine radiotherapy planning CTs is a particularly challenging task as respiratory motion, cardiac motion, as well as the varying extent of co-morbidities can impair visualization and result in large inter-observer variation particularly of small structures, such as the valves or coronary arteries (78). Even when sub-structures have been prospectively contoured, important variations can be seen due to the different guidelines available, or different interpretation of existing guidelines (79). For example, Thor et al. (80) have analyzed the heart doses reported in the RTOG 0617 clinical trial and demonstrated that inconsistencies in delineation led to a significant underestimation of cardiac exposure. They concluded that auto-contouring (e.g., using deep-learning to segment the whole heart) could increase the quality of clinical trials and the reliability of dose-toxicity associations explored in secondary analyses.

Several groups have developed auto-segmentation tools, using either atlas-based (81, 82) or machine-learning approaches (83) to address this issue. The performance of these auto-contouring solutions continues to improve but is affected by uncertainties in the manual contours used for training/validation. Another avenue that some authors have pursued is to use motion compensation to improve the quality of planning CTs and reduce contour uncertainties. Even though the motion of heart sub-structures due to heartbeat is reportedly small (typically <5 mm) (84), the heart can move 5-20 mm due to respiration. With the adoption of 4DCT worldwide, the use of different reconstruction techniques may add uncertainty to heart sub-structure segmentation. A potential solution to mitigate this issue is the use of motion compensated (mid-position) reconstructions which have been shown to reduce inter- and intra-observer contouring variations for organs at risk in patients with lung cancer (85). Moreover, Abravan et al. evaluated cardiac calcification detection in different phases of the respiratory cycle and found better detection in the extreme position of the respiratory cycle (69).

Novel methodologies can be used to generate evidence about the effectiveness of new treatments such as heart-sparing radiotherapy where RCT data will not exist. The robustness of biases can be assessed by employing probabilistic bias analysis, an approach that systematically assesses the extent of potential confounders (86). Utilizing new approaches such as causal inference, in which expert assumptions about causal mechanisms of outcomes are directly incorporated into statistical models using observational data, may further help with minimizing the effect of confounding. For example, the study discussed previously in which positional set-up errors are used to infer changes in heart dose, uses an instrument variable approach that can be compared to a natural randomized experiment (45). Such studies increase confidence that the observed differences in survival are caused by the differences in heart dose and are not merely associations with different underlying causes.

### How Results From Real-World Data Could Be Utilized in the Clinic?

The ultimate aim of RWD studies in the field of RIHD is to introduce and apply a dose limit to a defined anatomical area of the heart as part of the treatment planning process. If the evidence on sparing of anatomical regions of the heart is equivocal, equipoise can be argued and randomized designs such as pragmatic point-of-care or simple trial could be used (87). These approaches are designed to evaluate the effectiveness of interventions as an embedded part of routine practice, and are intermediate between RCTs and quasi-experimental studies. They aim to preserve a high degree of internal validity while reducing some of the disadvantages of conventional RCTs. However, as argued above, evidence in the field of radiotherapy-induced cardiac toxicity for patients with lung cancer is accumulating and it is becoming more difficult to argue equipoise. In this context, non-randomized quasi-experimental designs could instead be used in which specific outcome measures before and after a new clinical intervention is implemented are compared (76).

### Use of Rapid Learning as a Methodology to Assess the Impact of Heart Dose on Survival

**Figure 1:**
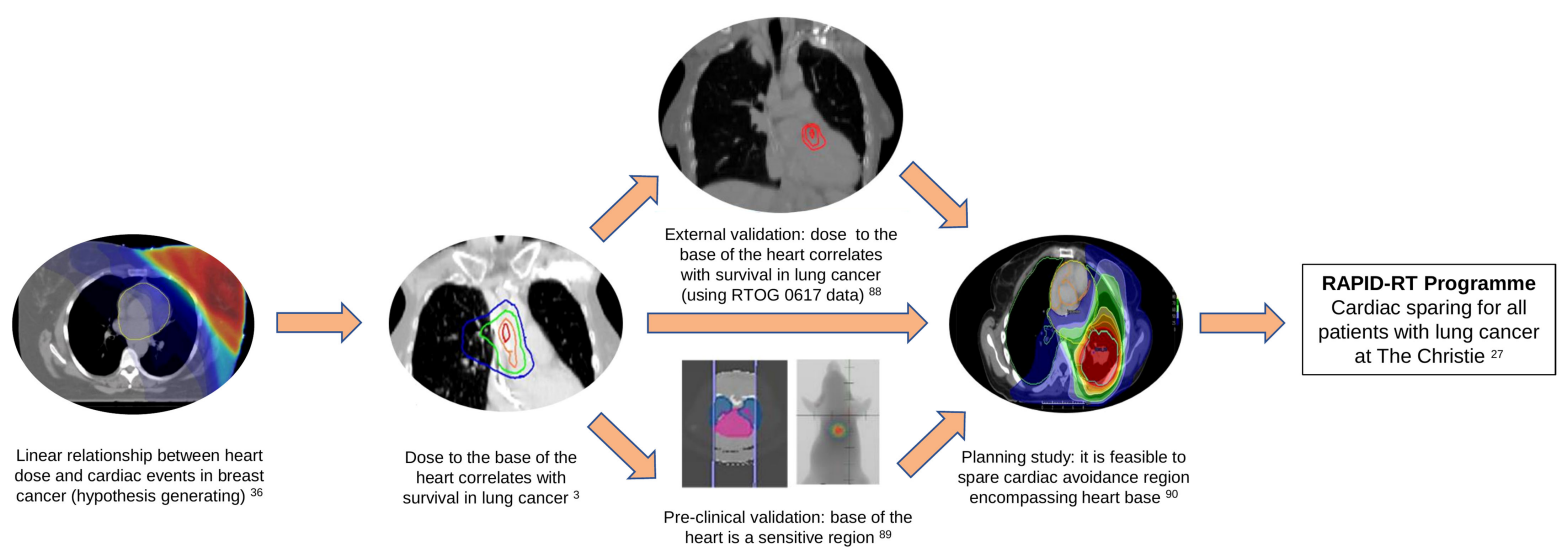
Evidence leading to the development of the RAPID-RT programme of research. In 2013, Darby et al. reported a linear relationship between mean heart dose and major coronary events in patients with breast cancer treated with radiotherapy (36). Mcwilliam et al, in 2017, showed there is a strong correlation between dose received by the base of the heart and overall survival in patients with lung cancer (3). These findings were externally validated using data from RTOG 0617 trial (88). A pre-clinical study further validated that the base of the heart is a sensitive region (reverse translation) (89). A feasibility planning study demonstrated that it is possible to spare cardiac avoidance region (located at the base of the heart) on previous Manchester studies (90). The RAPID-RT programme, funded by the National institute for health research (NIHR), will evaluate the impact of the introduction of a dose limit to the cardiac avoidance region in all patients with stage II-III lung cancer treated with curative-intent radiotherapy at The Christie NHS Foundation Trust in Manchester, UK (27).

Recent RWD studies (3, 9, 19, 20), have shown that incidental dose to the base of the heart increases the risk of early mortality in patients with lung cancer. A cardiac avoidance region was defined based on our previous studies encompassing structures located at the base of the heart including superior vena cava, right atrium, aortic root, and proximal segments of the coronary arteries. It is hypothesized that the cardiac toxicity is a result of damage done to the conduction system and the coronary arteries through inflammation, fibrosis, or ischemia. The RAPID-RT programme recently funded by the UK National Institute for Health Research (NIHR) is a large-scale research programme which will evaluate a change in the radiotherapy protocol to spare a cardiac-avoidance region in patients with stage II-III lung cancer treated with curative-intent radiotherapy at The Christie NHS Foundation Trust in Manchester, UK (27). A summary of the evidence that has fed into this programme of research is summarized in **Figure 1**. This change in the radiotherapy protocol is expected to increase patients’ short-term survival by 10-20%. Nevertheless, changing treatment delivery to spare the heart avoidance region without compromising tumor coverage may increase the dose to other organs at risk nearby, primarily the lungs, which in return may increase the risk of other toxicities, such as radiation pneumonitis (27). The programme will use a quasi-experimental interrupted time series design, with multiple cycles of learning, to assess the impact of the introduction of a dose limit to the cardiac avoidance region on survival and other toxicities using RWD. Related studies will assess the quality of the evidence derived from the rapid-learning methodology and how either it, or the methodological RWD approach, can be used to contribute to evaluate the impact of changes made to other aspects of radiotherapy pathway in other centers.

## Conclusion

In this review we demonstrated that high-quality RWD has the potential to provide robust evidence in the field of RIHD. Although RCTs are generally not practical for the evaluation of cardiac toxicity, emerging evidence and new methodologies using RWD are providing an alternative to the classical RCTs. The RAPID-RT study will use RWD to assess the clinical impact of introducing a new cardiac avoidance region dose constraint with the aim of reducing the risk of RIHD and improving survival.

## Author Contributions

All authors listed have made a substantial, direct, and intellectual contribution to the work and approved it for publication.

## Funding

This work did not receive any specific grant from funding agencies in the public, commercial, or not-for-profit sectors.

## Conflict of Interest

The authors declare that the research was conducted in the absence of any commercial or financial relationships that could be construed as a potential conflict of interest.

## Publisher’s Note

All claims expressed in this article are solely those of the authors and do not necessarily represent those of their affiliated organizations, or those of the publisher, the editors and the reviewers. Any product that may be evaluated in this article, or claim that may be made by its manufacturer, is not guaranteed or endorsed by the publisher.
